# Are Altered Knee Joint Biomechanics Associated with Future Post-Traumatic Osteoarthritis Outcomes? A Systematic Review and Meta-Analysis of Longitudinal Studies

**DOI:** 10.1007/s40279-025-02288-1

**Published:** 2025-08-05

**Authors:** Matthew Savage, Adam G. Culvenor, Michael Hedger, April-Rose Matt, Michael J. M. O’Brien, Rachael M. McMillan, Alysha De Livera, Benjamin F. Mentiplay

**Affiliations:** 1 La Trobe Sport and Exercise Medicine Research Centre, School of Allied Health, Human Services and Sport, La Trobe University, Melbourne, VIC Australia; 2 Australian IOC Research Centre, La Trobe University, Melbourne, VIC Australia; 3 School of Medicine, Deakin University, Waurn Ponds, VIC Australia; 4 Mathematics and Statistics, School of Computing, Engineering and Mathematical Sciences, La Trobe University, Melbourne, VIC Australia; 5 Sport and Exercise Science, School of Allied Health, Human Services and Sport, La Trobe University, Melbourne, VIC Australia

## Abstract

**Background:**

Post-traumatic knee osteoarthritis affects an estimated one in two people within a decade of traumatic knee injury. While altered biomechanics in older adults are associated with the onset and progression of insidious-onset knee osteoarthritis, the relationship between biomechanics and post-traumatic osteoarthritis is less clear.

**Objective:**

We aimed to evaluate associations between knee biomechanics and future structural and symptomatic outcomes post-surgery for traumatic knee injuries.

**Methods:**

We systematically searched MEDLINE, EMBASE, Scopus, CINAHL, and SPORTDiscus from inception until May 2025. The eligibility criteria were studies that: (1) included participants post-surgery for traumatic knee injuries; (2) assessed knee biomechanics (kinetics, kinematics) during dynamic tasks (e.g. walking, hopping); and (3) reported longitudinal associations between early knee biomechanics and future post-traumatic osteoarthritis outcomes, including joint structure (imaging) or symptoms (patient-reported outcomes). Meta-analyses were completed where possible, with the remaining studies synthesised narratively due to heterogeneity precluding meta-analysis.

**Results:**

We included 18 studies (structure = 12, symptoms = 6); 17 following anterior cruciate ligament reconstruction and one post-meniscectomy. Meta-analysis of three studies examined the association between patellofemoral contact force up to 1-year post-anterior cruciate ligament reconstruction and future cartilage structure at 1–5 years, assessed via T2 relaxation times and progression of cartilage defects on magnetic resonance imaging. Lower patellofemoral contact force was associated with worse future trochlear cartilage structure (*r* = − 0.48, 95% confidence interval − 0.63, − 0.31; *I*^2^ = 0%), but the association with patellar cartilage was not significant (*r* = − 0.09, 95% confidence interval − 0.30, 0.12; *I*^2^ = 0%). A meta-analysis of three studies found no relationship between joint kinetics (e.g. knee flexion moment or knee adduction moment) and future structural outcomes in the tibiofemoral compartment, including T1 rho relaxation times, cartilage defects on magnetic resonance imaging and radiographic osteoarthritis. Narrative synthesis of other studies found that lower kinetic measures (e.g. knee flexion moment, knee adduction moment) were associated with worse future trochlear cartilage, but relationships with patellar cartilage and tibiofemoral joint structure were inconsistent. For symptoms, although time post-surgery appears to influence associations with mechanical loading, lower measures of frontal plane kinetics (e.g. knee adduction moment, medial ground reaction force) were associated with better future symptoms regardless of the timepoint.

**Conclusions:**

Underloading of the patellofemoral joint within the first year post-anterior cruciate ligament reconstruction is associated with worse patellofemoral cartilage, a pattern not observed in the tibiofemoral joint. Clinicians should consider optimising loading interventions and addressing modifiable biomechanical alterations post-surgery to preserve cartilage health and reduce symptoms.

**Protocol Registration:**

PROSPERO: CRD42024504099.

**Supplementary Information:**

The online version contains supplementary material available at 10.1007/s40279-025-02288-1.

## Key Points

**Table:** 

Underloading of the patellofemoral joint up to 1-year post-anterior cruciate ligament reconstruction is a potential risk factor for future post-traumatic patellofemoral osteoarthritis at 1–5 years post-anterior cruciate ligament reconstruction.
The evidence for the relationship between mechanical loading and post-traumatic tibiofemoral osteoarthritis is inconsistent regardless of the timepoint they were assessed.
The evidence for the relationship between mechanical loading and future symptoms is inconsistent: lower vertical ground reaction force in the first 6 months post-surgery may be associated with worse future symptoms at 1 year, while lower measures of frontal plane kinetics (e.g. knee adduction moment, medial ground reaction force) were associated with better future symptoms regardless of the timepoint they were assessed.

## Introduction

Post-traumatic knee osteoarthritis (OA) affects an estimated one in two people within a decade of traumatic knee injury, such as anterior cruciate ligament (ACL) rupture or meniscal tear [1], irrespective of whether they undergo surgical or non-surgical management [2]. Given that these injuries are most common during adolescence and young adulthood, post-traumatic OA typically develops before the age of 40 years, resulting in more years lived with disability than insidious-onset OA in older adults [3]. Post-traumatic knee OA is characterised by structural joint changes on imaging and/or symptoms. Although most post-traumatic OA research has focused on structural outcomes [2], knee symptoms (e.g. pain) drive the large personal and societal burden of OA and motivate patients to seek healthcare [4].

The aetiology underpinning the onset and progression of post-traumatic OA is not fully understood [5], but is thought to be related to: (i) the initial inflammatory response and intra-articular haemarthrosis becoming chronic [6]; (ii) concomitant injury to joint structures (e.g. articular cartilage, subchondral bone, synovium) [6]; and (iii) altered knee joint biomechanics that occur following injury and are not fully restored with surgery (e.g. ACL reconstruction [ACLR], meniscal repair) [7, 8]. Given the rising incidence of surgery for traumatic knee injuries [9, 10], and evidence that surgical interventions alone may not reduce the risk of developing OA [11], a deeper understanding of how post-surgical factors contribute to the onset and progression of post-traumatic OA is needed.

Aberrant biomechanics linked to elevated stress on joint cartilage, such as a greater knee adduction moment (KAM) and varus thrust, are commonly observed in older adults with insidious-onset knee OA, and have been associated with both structural and symptomatic disease onset and progression [12–14]. In post-traumatic OA, the relationship between biomechanics and structural and symptomatic outcomes is less clear. Cross-sectional studies have shown that the relationship between biomechanics and OA outcomes differs between patients with insidious-onset and post-traumatic knee OA [15, 16]. Recently, post-traumatic OA has been linked with biomechanical patterns of joint underloading [17, 18], directly contrasting the elevated joint loads typically observed with insidious-onset OA. Notably, recent findings suggest that as early as 6–12 months post-ACLR, individuals exhibit less dynamic ground reaction force (GRF) profiles, resembling those of patients with Kellgren–Lawrence Grade 2 knee OA, with lower peak vertical GRF and higher midstance loading, suggesting the presence of joint underloading [19]. However, increased frontal plane loading, such as higher KAM, has been associated with early cartilage degeneration, suggesting that specific frontal plane overloading may exacerbate disease progression [20, 21], reflecting the complex interplay of biomechanical factors and future OA.

Given the modifiable nature of altered knee joint biomechanics, understanding the link between biomechanics and longitudinal changes in structural and symptomatic outcomes may help guide post-traumatic OA prevention strategies [22]. Therefore, we aimed to systematically synthesise the evidence of the association between knee joint biomechanics and future joint structural and symptomatic outcomes in individuals following surgery for a traumatic knee injury. We hypothesised that joint underloading would be associated with poorer structural and symptomatic outcomes.

## Methods

This systematic review was reported according to Preferred Reporting Items for Systematic Reviews and Meta-Analyses (PRISMA) guidelines [23], and was prospectively registered (PROSPERO: CRD42024504099).

### Search Strategy

We performed a systematic search in September 2021 (updated May 2024 and May 2025) with no restriction on publication year in MEDLINE, EMBASE, Scopus, CINAHL and SPORTDiscus. The search combined Medical Subject Headings (MeSH) terms and keywords related to knee surgery, biomechanics, and measures of knee joint structure or symptoms (Appendix A of the Electronic Supplementary Material [ESM]). The reference lists of included articles were screened for additional relevant publications.

### Eligibility Criteria

Peer-reviewed longitudinal studies were eligible if they: (i) were published in English; (ii) included human participants with a history of knee surgery following traumatic injury; (iii) assessed lower-limb biomechanics during dynamic tasks (e.g. walking, running, hopping); and (iv) reported longitudinal associations between early knee joint relevant biomechanics (or changes in relevant biomechanics over time) and future post-traumatic OA outcomes, including future structural outcomes assessed using imaging (e.g. X-ray, magnetic resonance imaging [MRI]) or symptomatic outcomes assessed using patient-reported outcomes. We excluded case studies, non-original data studies (e.g. reviews, editorials) and abstracts, as well as studies examining exclusively knee arthroplasty or intra-articular fracture populations. Studies examining only biomechanics specifically related to other joints (e.g. hip, ankle), or investigating static alignment or loading that were not assessed biomechanically (e.g. step counts), were also excluded. Two authors (one of MS, MH, AC, RM, MO, AM) independently screened titles, abstracts and relevant full texts for eligibility. Disagreements were discussed until a consensus was reached as reported previously [24].

### Data Extraction

The following information was extracted from the included studies independently by two authors (two of MH, BM, MS and conflicts resolved in consultation with AC) using a customised data extraction spreadsheet. Participant characteristics (number, age, sex, body mass index [BMI]), task assessed, biomechanical exposure variable(s), structural and/or symptomatic outcome measure(s), follow-up duration and effect size (and accompanying measure of uncertainty) for the relationship between biomechanical variables and future structural/symptomatic outcomes were recorded. Any relationships using uninvolved limb data reported alone (e.g. not included as part of the limb symmetry index) and pre-surgical biomechanical data were not extracted.

### Risk of Bias

Risk of bias was independently assessed by three reviewers (MH, BM, MS), with a fourth available to resolve disagreements (AC). The Quality in Prognosis Studies (QUIPS) tool (Appendix B of the ESM) assessed the risk of bias as recommended by the Cochrane Prognosis Methods Group [25]. The QUIPS tool assesses validity and sources of bias in prognostic factor studies across six domains: participation, attrition, prognostic factor measurement, outcome measurement, confounding, and statistical analysis and reporting. The QUIPS tool provides multiple prompting questions to guide reviewers to determine the potential risk of bias as either low, medium or high in each domain [25].

### Data Synthesis

Outcomes were separated into either structural or symptomatic outcomes. Structural outcomes were further grouped by the joint investigated (i.e. tibiofemoral, patellofemoral) and time post-surgery (within, or after, 12 months). For structural outcomes, we analysed the most commonly reported measures. For example, where studies reported multiple sub-regions, we included the whole region (e.g. medial tibia) in a meta-analysis and narrative synthesis. For symptom outcomes, when multiple measures were reported, we prioritised the Knee injury and Osteoarthritis Outcome Score (KOOS) for analysis, using either individual subscales or the aggregated KOOS_4_, when available. This prioritisation was based on the KOOS being the most frequently reported outcome measure across studies, facilitating standardised synthesis and comparison of results. We defined loading as a term encompassing any kinetic measure (e.g. joint contact force or pressure, joint moments or ground reaction forces). Throughout this article, we use the term loading to: (i) cluster multiple kinetic variables, providing an overall picture of knee loading; and (ii) describe and aid interpretation of the results of our meta-analyses involving individual kinetic variables.

We performed random-effects meta-analyses for each group of outcomes where appropriate (trochlear cartilage, patellar cartilage, tibiofemoral structure, symptoms). When heterogeneity precluded the ability to undertake a meta-analysis, findings were summarised using best-evidence narrative synthesis [26]. Where multiple reports from the same cohort were identified, we included the study with the largest sample for meta-analysis. For the narrative synthesis, we included multiple reports from overlapping cohorts when they provided unique contributions (e.g. different biomechanical exposures, different outcomes). A cohort overlap was noted, and reports were grouped by research group or cohort in Sect. 3 for transparency [26]. If studies reported results for both the index limb and comparisons with the contralateral limb (e.g. side-to-side differences, limb symmetry index), we included the index limb. If studies reported multiple variations of biomechanical variables (e.g. the same variable at various points of the gait cycle), data relating to the first peak were extracted for meta-analysis, but both results were included in the narrative synthesis. Authors of relevant studies were contacted to provide additional information, such as raw data, to facilitate inclusion in meta-analysis. Where possible, following Cochrane guidelines [26], effect estimates were converted and standardised to common scales, to facilitate inclusion in meta-analysis (see Appendix C of the ESM for details). We assessed for heterogeneity by: (i) using the I^2^ statistic in meta-analyses [26]; and (ii) comparing methodologies (including study population, biomechanics assessed, structural/symptom outcomes, post-surgery and follow-up timepoints) in the narrative synthesis. Effect sizes for Pearson’s r correlation coefficients were interpreted as small (0.1–0.3), medium (0.3–0.5) or large (> 0.5) [27]. Effect sizes for standardised mean differences (SMDs) were interpreted as small (< 0.4), moderate (0.4–0.7), or large (> 0.70) [27]. Data analyses were performed using the “metacor” and “meta” packages in R-Studio (version 4.4.0) [28].

**Table 1:** Included study characteristics

Study	Location	Participants, *n*	Male, *n* (%)	Age, mean ± SD, years	BMI, mean ± SD, kg.m^2^	Task	Biomechanical metric	Biomechanics post-surgery timepoint	Outcome	Follow-up outcome timepoint	Effect estimate
*Studies that included structural outcomes*											
Capin et al. [29]	Delaware	26	16 (62%)	23 ± 7	26.8^a^	Walking preferred speed	KFM^b^ Peak quadriceps muscle forces^b^ KFA^b^	3 months	**T2 relaxation time** Trochlea^b^	6 months	Pearson’s correlation R^2^
Erhart-Hledik et al. [30]	Stanford	17	5 (29%)	30 ± 7	23.5 ± 2.7	Walking preferred speed	KFM KAM Total knee joint moment	Δ 2–8 years	**Cartilage thickness ratio (medial:lateral)** Femur	Δ 2–8 years	Pearson’s correlation
Evans-Pickett et al. [31]	UNC	26	13 (50%)	22 ± 4	24.2 ± 3.5	Walking preferred speed	KFM KAM	6 months	**T1 rho** Weight bearing TFJ	12 months	Cohen’s d (95% CI)
Hall et al.^c^ [32]	University of Melbourne	70	61 (87%)	41 ± 5	27.2 ± 4.1	Walking preferred speed Walking fast pace	KFM KAM	3 months	**Cartilage volume** Medial tibia Patella **Cartilage defects** Medial tibiofemoral Patella	Δ 3 months to 2 years	Regression coefficient (95% CI) Simple regression (odds ratio)
Kumar et al [33]	UCSF	37	22 (60%)	31 ± 5	23.3 ± 2.2	Walking fixed speed 1.35 m/s	KAM	Δ Pre-op to 6 months Δ 6 months to 1 year	**T1 rho** Femur Tibia **T2 relaxation time** Femur Tibia	Δ pre-op to 6 months Δ 6 months to 1 year	Pearson’s correlation
Liao et al.^d^ [34]	UCSF	32	27 (55%)	29 ± 8	24.4 ± 3.5	Walking fixed speed 1.3 m/s	PFJ contact pressure	6 months	**T2 relaxation time** Patella Trochlea	3 years	Pearson’s correlation
Schache et al. [35]	University of Melbourne	32	20 (63%)	Worsening PFJ OA: 26 ± 5 No worsening: 2 6 ± 4	Worsening PFJ OA: 24.9 ± 3.1 No worsening: 24.8 ± 3.5	Forward hop	PFJ contact force	1 year	**Cartilage lesions** Patellofemoral	Δ 1–5 years	Risk ratio
Shimizu et al. [36]	UCSF	31	17 (55%)	31 ± 1	23.5 ± 0.4	Drop jump	KFM^e^ vGRF^e^ KFA^e^	6 months Δ 6 months to 3 years	**T1 rho** Femur Tibia	Δ 6 months to 3 years	β
Shimizu et al. [37]	UCSF	36	20 (56%)	32 ± 8	23.9 ± 2.5	Drop jump	KFM vGRF	6 months	**T1 rho** PHMED meniscus **T2 relaxation time** PHMED meniscus	Δ 6 months to 3 years	β
Teng et al [38]	UCSF	33	20 (61%)	31 ± 9	23.9 ± 2.7	Walking fixed speed 1.3 m/s	KFM vGRF KFA	6 months 1 year	**T1 rho** Femur Tibia **T2 relaxation time** Femur Tibia	Δ pre-op to 1 year Δ pre-op to 2 years	R^2^ β
Wellsandt et al.^f^ [39]	Delaware	14^e^	9 (64%)^e^	Non-OA: ^g^ 33 ± 11 OA: ^g^ 45 ± 6	Non-OA: ^g^ 28.1 ± 2.9 OA: ^g^ 25.2 ± 2.4	Walking preferred speed	Peak medial contact force^b^ KFM^b^ KAM^b^	6 months 1 year 2 years	**XR (KL)** OA or no OA (medial compartment TFJ)	5 years	Mean (SD)
Williams et al. [40]	Delaware	30	16 (53%)	23 ± 7	25.9 ± 3.8	Walking preferred speed	PFJ contact force KFM KFA	3 months	**T2 relaxation time** Patella Trochlea	2 years	Pearson’s correlation R^2^
*Studies that included symptom outcomes*											
Azus et al. [41]	UCSF	43	25 (58%)	30 ± 8	23.7 ± 2.6	Walking fixed speed 1.33 m/s	KFM vGRF Medial GRF	6 months	KOOS pain KOOS symptoms	1 year Δ 6 months to 1 year	Partial correlation
Erhart-Hledik et al. [42]	Stanford	16	5 (31%)	29 ± 7	23.7^a^	Walking preferred speed	KFM^e^ KAM^e^ Knee internal rotation moment^e^ KFA^e^ Varus tibial rotation angle^e^ Knee internal rotation angle^e^ Anterior displacement of the femur relative to the tibia^e^	2 years	KOOS pain KOOS QoL	Δ 2–8 years	Pearson’s correlation
Erhart-Hledik et al. [43]	Stanford	28	11 (39%)	29 ± 6	24.7 ± 3.5	Walking preferred speed	vGRF^e^	2 years	KOOS pain KOOS symptoms KOOS ADL KOOS sport/rec KOOS QoL	Δ 2–10 years	Pearson’s or Spearman’s correlation
Ithurburn et al. [44]	Ohio State	48	11 (23%)	18 ± 3	NR	Single-leg drop landing	KFA^e^ Trunk flexion^e^	At time of return to sport	KOOS pain KOOS ADL KOOS QoL	2 years after RTS	Odds ratio (95% CI)
Pietrosimone et al. [45]	UNC	25	13 (52%)	21 ± 4	23.7^a^	Walking preferred speed	vGRF^e^	6 months	KOOS pain KOOS symptoms KOOS ADL KOOS sport/rec KOOS QoL	1 year	R^2^
Titchenal et al.^d^ [46]	Stanford	26	15 (58%)	31 ± 6	24.8 ± 2.6	Walking preferred speed	Knee centre of rotation (AP) Knee centre of rotation (ML)	Δ 2–4 years	KOOS pain KOOS symptoms KOOS QoL	Δ 2–4 years Δ 2–8 years	Pearson’s correlation R^2^

*AP* anterior–posterior, *BMI* body mass index, *CI* confidence interval, *KAM* knee adduction moment, *KFA* knee flexion angle, *KFM* knee flexion moment, *KL* Kellgren–Lawrence, *KOOS* Knee injury and Osteoarthritis Outcome Score, *Medial GRF* medial ground reaction force, *ML* medial–lateral, *NR* not reported, *OA* osteoarthritis, *PFJ* patellofemoral joint, *PHMED* posterior horn of the medial meniscus, *QoL* quality of life, *RTS* return to sport, *SD* standard deviation, *TFJ* tibiofemoral joint, *vGRF* vertical ground reaction force, *XR* X-ray, Δ change in

^a^Calculated from available height and body mass data

^b^Limb value and side-to-side difference

^c^Arthroscopic partial meniscectomy (the other included studies are all from participants post-anterior cruciate ligament reconstruction)

^d^Demographic data on 49 baseline participants

^e^Side-to-side difference/limb symmetry index

^f^Demographic data on 14 participants at the 6-month follow-up timepoint

^g^At 6-month data collection

^h^Demographic data on 26 participants at baseline and the 4-year follow-up

Further details on the exposure and outcome in each study can be found in the Appendix D of the ESM

## Results

### Study Characteristics

**Fig. 1:**
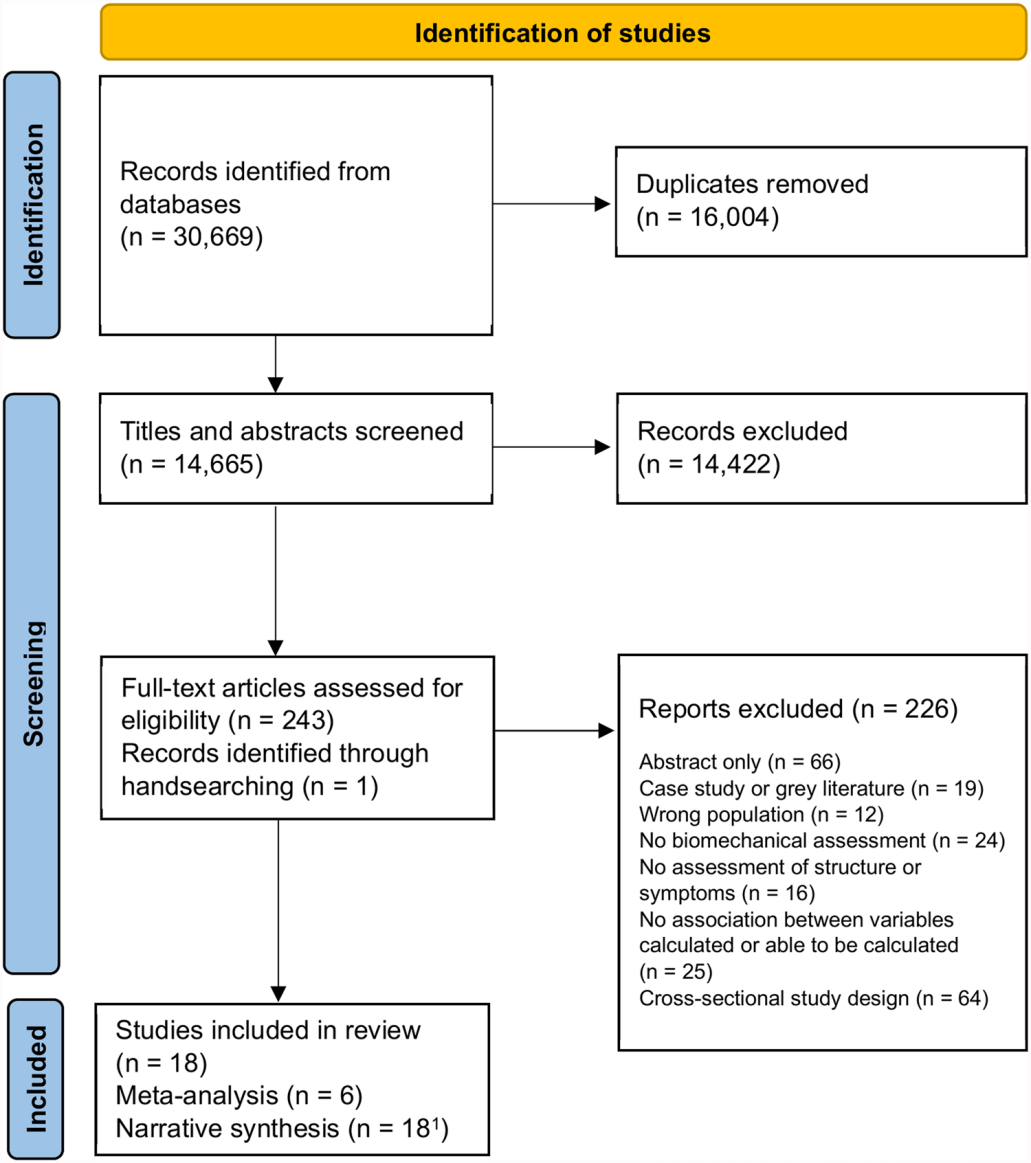
Flow diagram of included studies.^1^Studies included in the meta-analysis also reported results included in the narrative synthesis

Eighteen studies were included in our review (Fig. 1), 17 studies focused on individuals following ACLR and one study evaluated individual’s post-partial meniscectomy (Table 1). Most studies assessed walking biomechanics (*n* = 14), while others assessed drop landings (*n* = 3) and a forward hop (*n* = 1). The biomechanical variables used varied widely and often included multiple discrete values from within each variable (e.g. first peak, second peak, impulse). Sixteen studies examined loading using kinetic variables, with the most common being knee flexion moment [KFM] (*n* = 9), KAM (*n* = 6), vertical GRF [vGRF] (*n* = 6) and patellofemoral joint contact force or pressure (*n* = 3). Seven studies assessed kinematics, with knee flexion angle (KFA) the most frequently investigated (*n* = 4). Twelve studies evaluated the association between a biomechanical measure (or a change in biomechanical measure over time) and future structural outcomes [29–40]. Four studies investigated only structural outcomes in the patellofemoral joint [29, 34, 35, 40] and seven studies investigated structural outcomes only in the tibiofemoral joint [30, 31, 33, 36–39], while one study examined both [32]. The structural outcomes assessed were: cartilage quality [T1 rho and/or T2 cartilage relaxation times] (*n* = 8), cartilage thickness, volume and/or defects assessed by MRI (*n* = 3), and radiographic OA using the Kellgren–Lawrence classification (*n* = 1). Six studies examined the association between a biomechanical measure (or change in biomechanical measure over time) and future symptoms [41–46]. These studies all assessed symptoms using the KOOS (*n* = 6).

### Risk of Bias

Across the 18 studies, 5 (28%) had a low risk of bias for study participation, none (0%) had a low risk of bias for study attrition, 18 (100%) had a low risk of bias for prognostic factors, 17 (94%) had a low risk of bias for outcome measurement, 8 (44%) had a low risk of bias for confounding and 12 (67%) had low risk of bias for analysis (Appendix E of the ESM).

### Longitudinal Association Between Loading Measures and Future Structural Outcomes in the Patellofemoral Joint

**Fig. 2:**
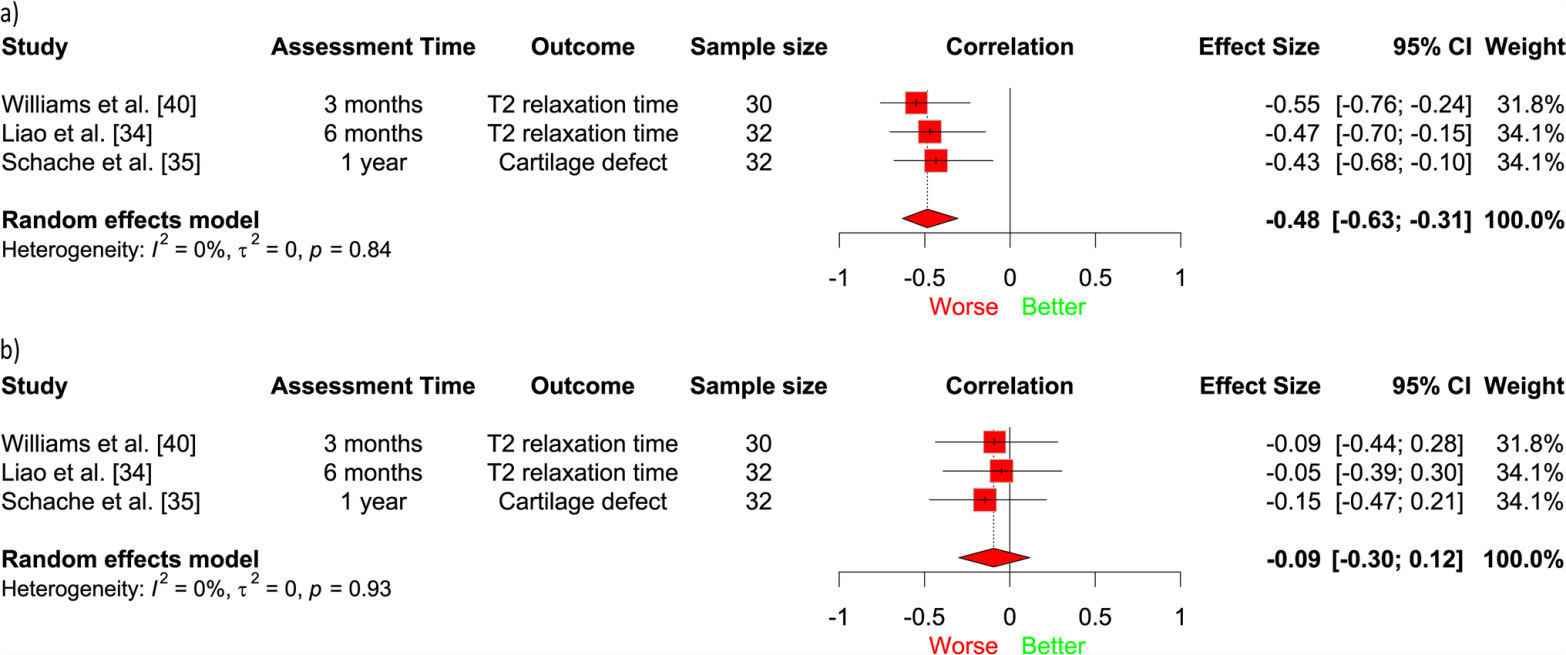
Association between patellofemoral contact force/pressure and structural outcomes in the: **a)** trochlea and **b)** patella. Results to the *left of the line* of no effect indicate that underloading is associated with worse future cartilage structure. *CI* confidence interval

**Fig. 3:**
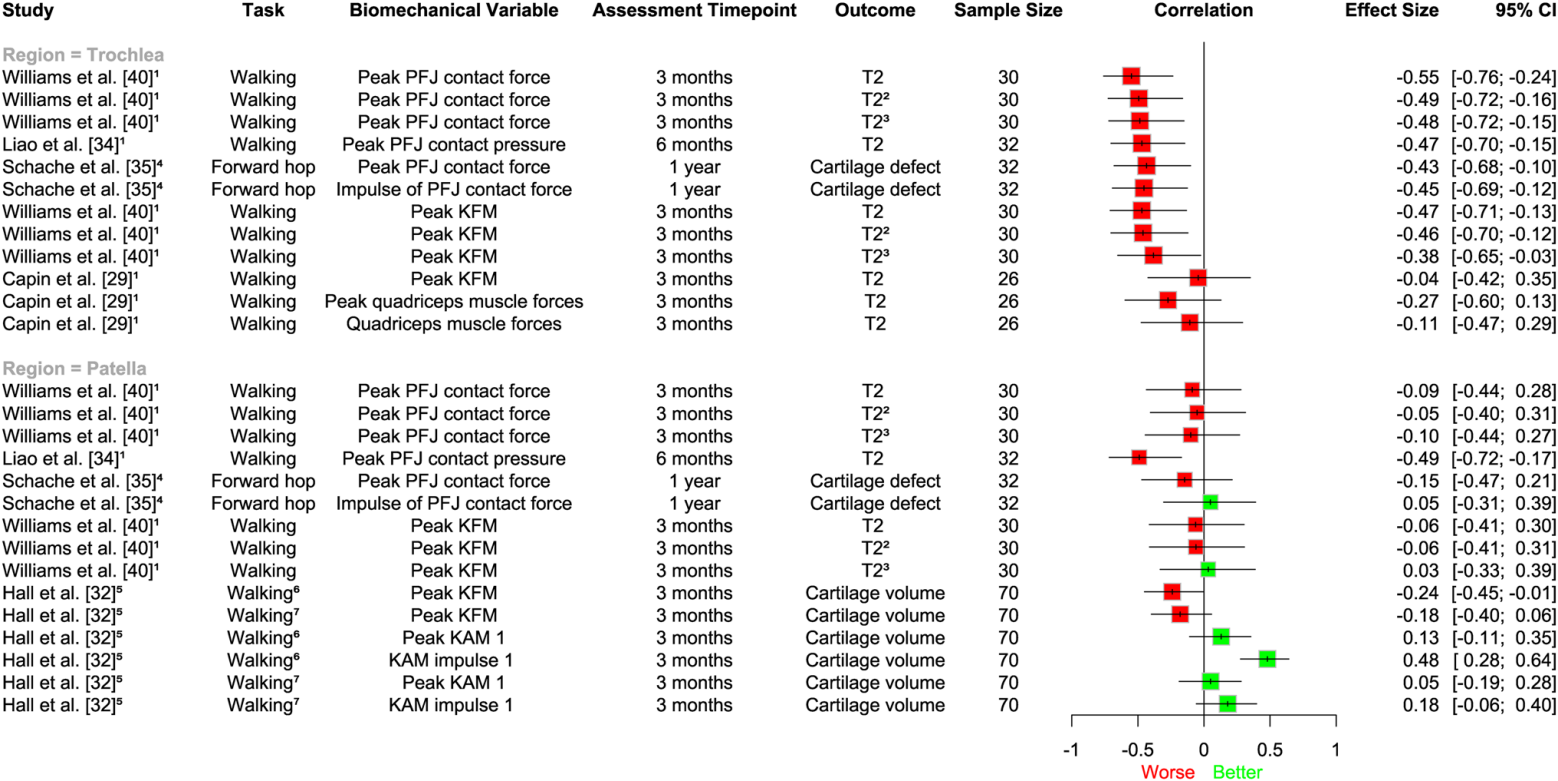
Descriptive plot of individual study results showing the association between lower magnitudes of joint load and cartilage outcomes in the patella and trochlea. Results to the left of the line of no effect indicate that lower loading is associated with worse future cartilage structure. *CI* confidence interval, *KAM* knee adduction moment, *KFM* knee flexion moment, *PFJ* patellofemoral joint, 1 = first half of stance phase, ^1^Pearson’s correlation coefficient; ^2^deep cartilage; ^3^superficial cartilage; ^4^point-biserial correlation; ^5^regression coefficient; ^6^preferred speed walking; ^7^fast speed walking

A meta-analysis of three studies (94 participants) [34, 35, 40] demonstrated that lower patellofemoral contact force/pressure during 3 months to 1-year post-ACLR was associated with worse trochlear cartilage structure at 1–5 years post-ACLR (medium effect size; *r* = − 0.48, 95% CI − 0.63, − 0.31; *I*^2^ = 0%; Fig. 2a), specifically increased T2 relaxation times and progression of cartilage defects on MRI. We did not find a statistically significant longitudinal association between lower patellofemoral contact force/pressure and future patellar cartilage structure, assessed via the same outcomes (*r* = − 0.09, 95% CI − 0.30 to 0.12; *I*^2^ = 0%; Fig. 2b). Similar to our meta-analyses, findings from other studies in our narrative synthesis [29, 34, 35, 40] indicate that lower magnitudes of joint load at 3 months to 1-year post-surgery (e.g. patellofemoral contact force, KFM, KAM, quadriceps muscle forces) seem to be associated with worse trochlear cartilage at 6 months to 5 years post-ACLR; however, results were more inconsistent for patellar cartilage (Fig. 3).

### Longitudinal Association Between Loading Measures and Structural Outcomes in the Tibiofemoral Joint

**Fig. 4:**
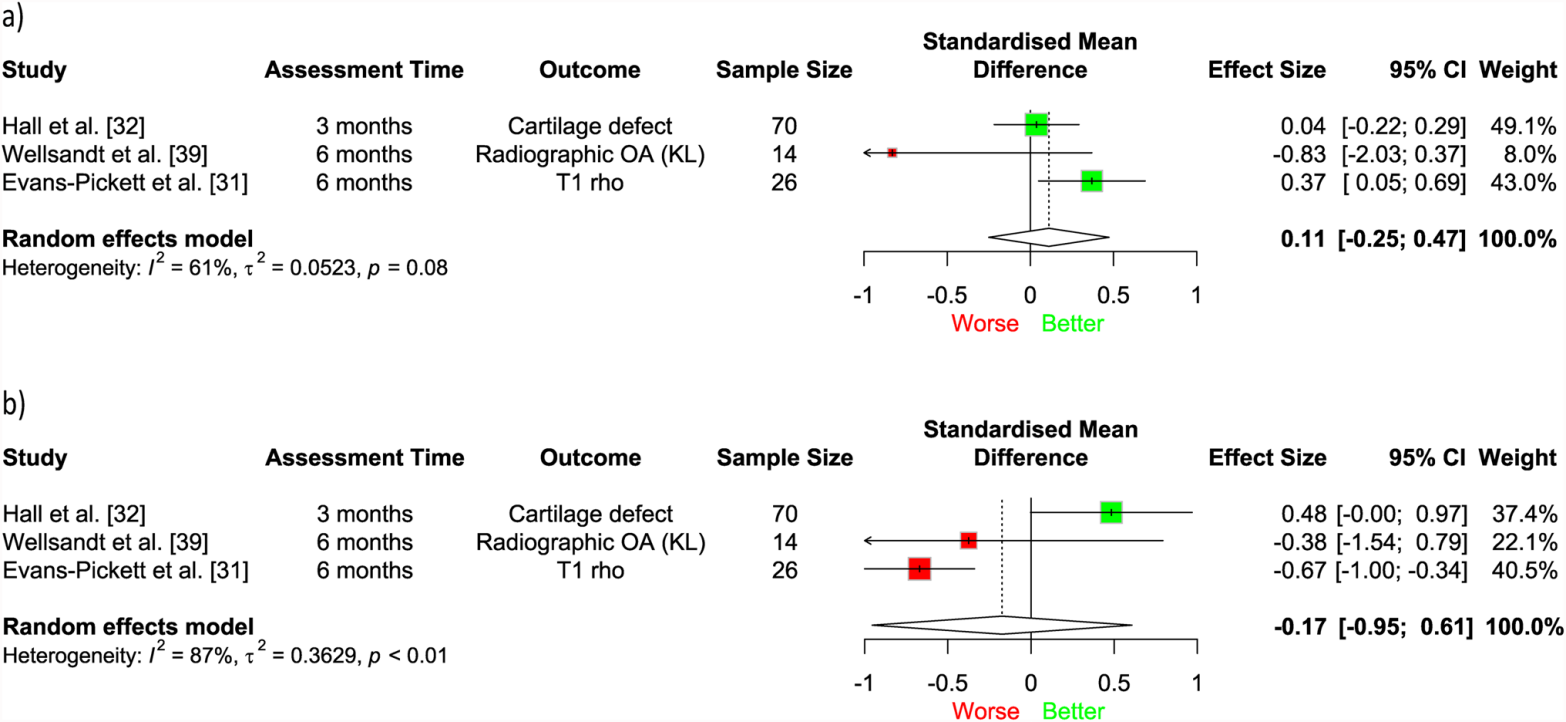
Association between **a)** knee flexion moment and **b)** knee adduction moment and structural outcomes in the tibiofemoral joint. Results to the left of the line of no effect indicate that lower loading is associated with worse future joint structure. *CI* confidence interval, *KL* Kellgren–Lawrence, *OA* osteoarthritis

**Fig. 5:**
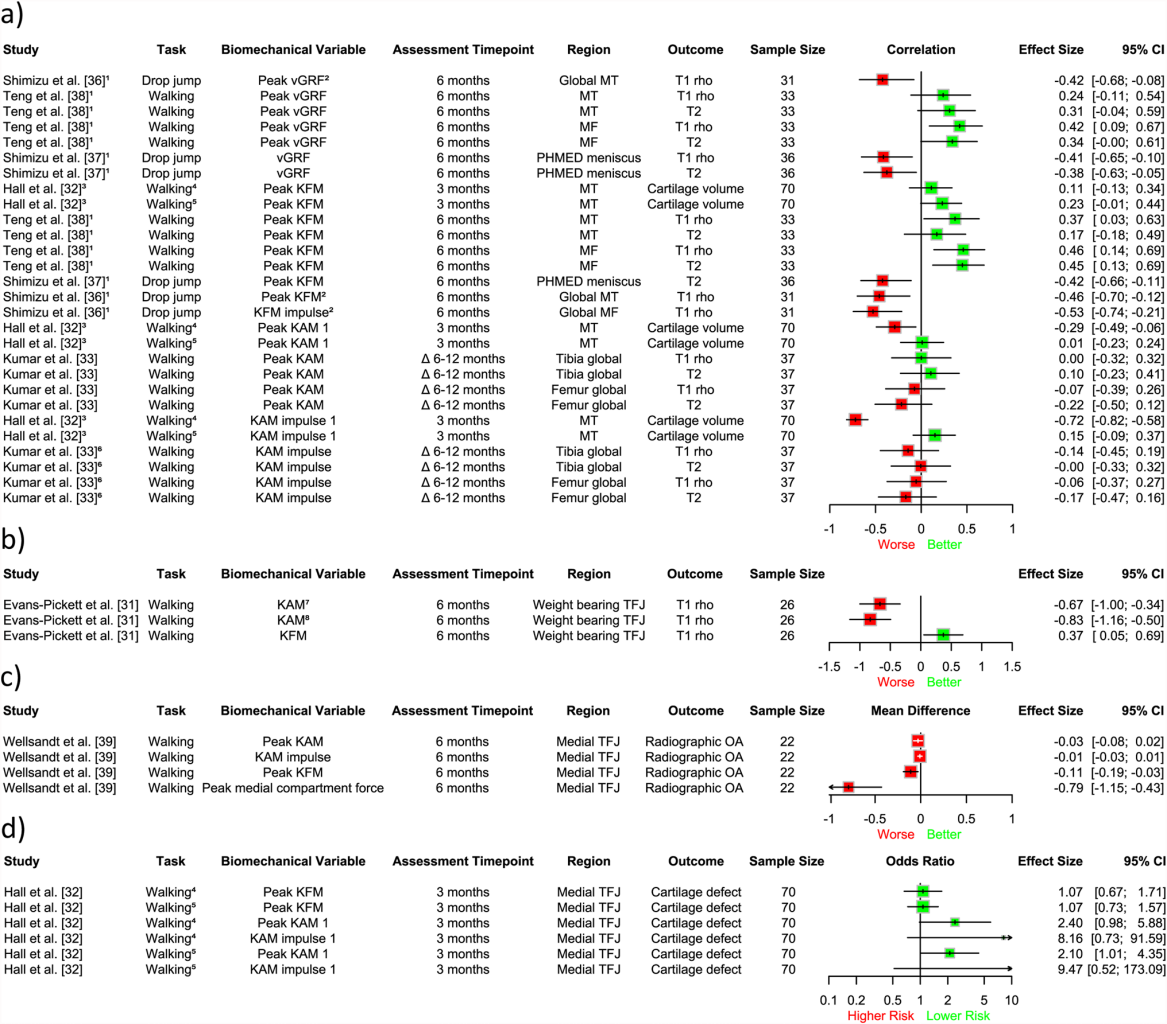
Descriptive plot of individual study results showing the association between lower magnitudes of joint load < 12 months post-surgery and structural outcomes in the tibiofemoral joint. **a)** Beta, regression and Pearson’s correlation coefficients; **b)** Cohen’s d effect size; **c)** mean differences; and **d)** odds ratios. Results to the left of the line of no effect indicate that lower loading is associated with worse future structural outcomes. 1 = first half of stance phase, *CI* confidence interval, *KAM* knee adduction moment, *KFM* knee flexion moment, *MF* medial femur, *OA* osteoarthritis, *MT* medial tibia, *PHMED* posterior horn of the medial meniscus, *TFJ* tibiofemoral joint, *vGRF* vertical ground reaction force, Δ change, ^1^beta coefficient; ^2^side-to-side difference; ^3^regression coefficient; ^4^preferred speed walking; ^5^fast speed walking; ^6^Pearson’s correlation coefficient; ^7^11–47% of stance phase; ^8^59–95% of stance phase

**Fig. 6:**
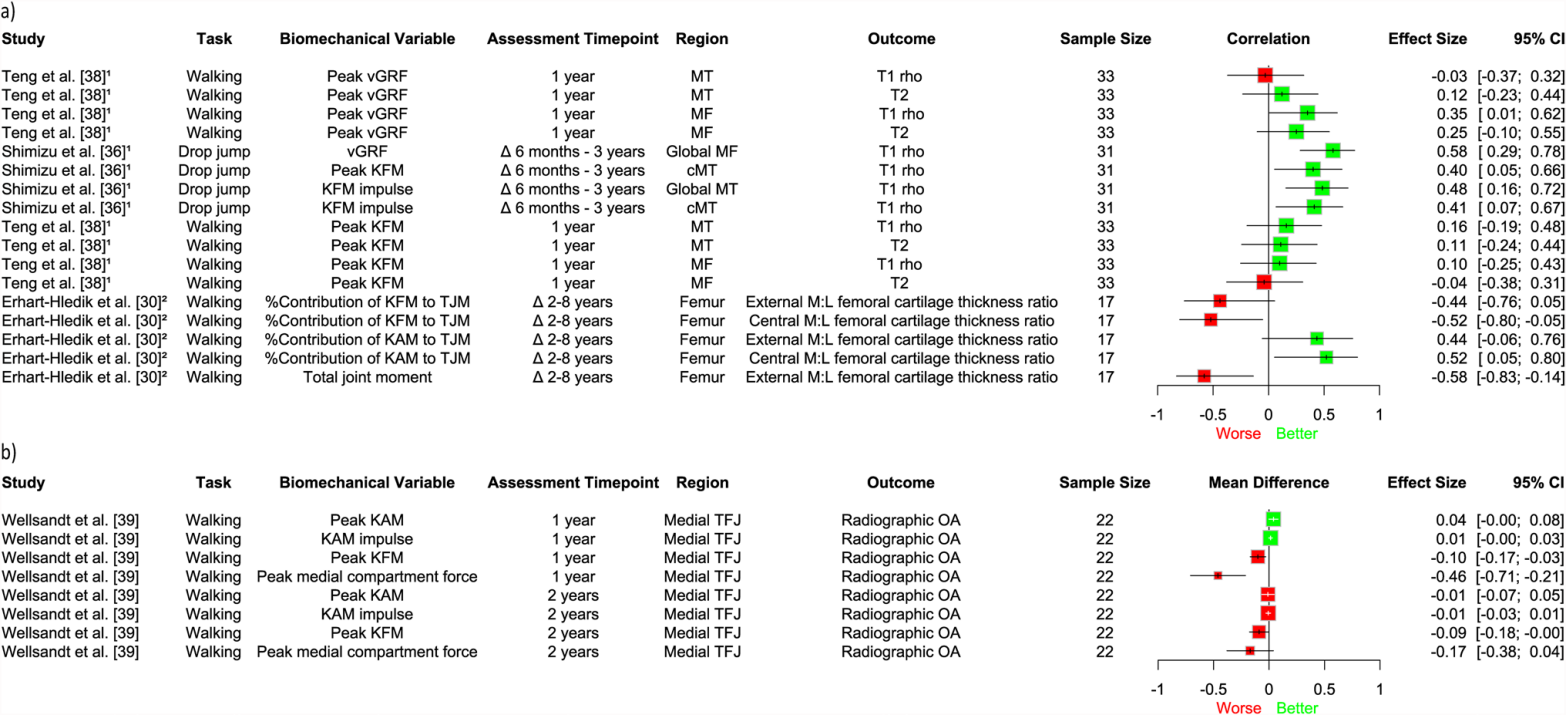
Descriptive plot of individual study results showing the association between lower magnitudes of joint load > 12 months post-surgery and structural outcomes in the tibiofemoral joint. **a)** Beta and Pearson’s correlation coefficients and **b)** mean differences. Results to the left of the line of no effect indicate that lower loading is associated with worse future structural outcomes. *CI* confidence interval, *cMT* central medial tibia, *KAM* knee adduction moment, *KFM* knee flexion moment, *MF* medial femur, *M:L* medial to lateral, *MT* medial tibia, *OA* osteoarthritis, *TFJ* tibiofemoral joint, *TJM* total joint moment, *vGRF* vertical ground reaction force, Δ change, ^1^beta coefficient; ^2^Pearson’s correlation coefficient

A meta-analysis of three studies (110 participants) [31, 32, 39] found no significant longitudinal association between KFM or KAM during 3–6 months post-surgery and future structural outcomes in the tibiofemoral joint at 3 months to 5 years (SMD = 0.11, 95% CI − 0.25, 0.47, *I*^2^ = 61%, Fig. 4a; SMD = − 0.17, 95% CI − 0.95, 0.61, *I*^2^ = 87%, Fig. 4b, respectively), assessed through T1 rho relaxation times, cartilage defects on MRI and radiographic OA. Furthermore, our narrative synthesis found inconsistent evidence for the longitudinal association between knee joint loading variables (including vGRF, KFM, KAM, total knee joint moment, medial compartment force) and future structural outcomes in the tibiofemoral compartment [30–33, 36–39], regardless of the measure of effect (e.g. correlation coefficients, regression coefficients, Cohen’s *d*, mean differences, odds ratios) or whether they were assessed in the first 12 months post-surgery (Fig. 5), or > 12 months post-surgery (Fig. 6).

### Longitudinal Association Between Loading Measures and Symptom Outcomes

**Fig. 7:**
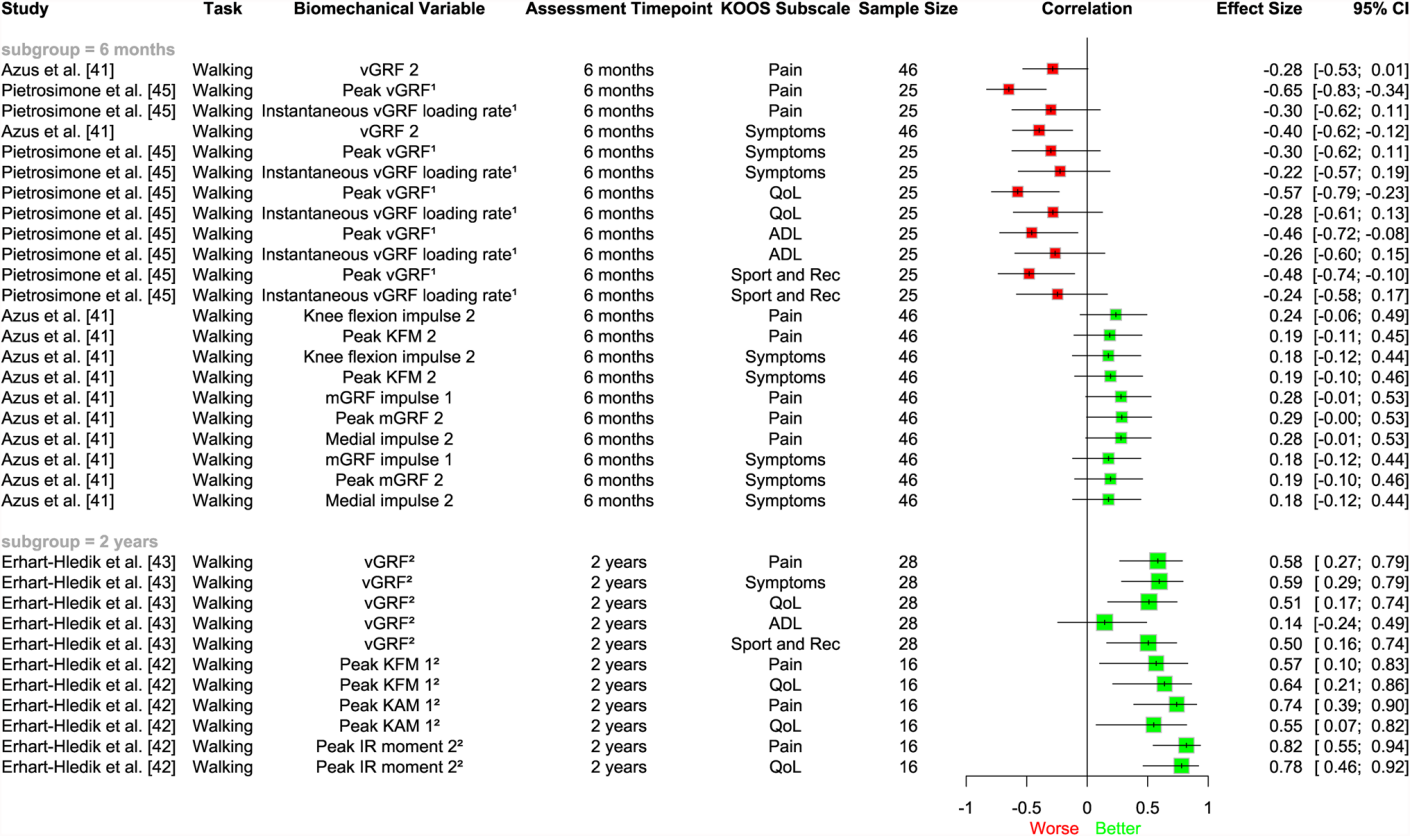
Descriptive plot of individual study results showing the association between lower magnitudes of joint load post-surgery and patient-reported outcomes. Results to the *left of the line* of no effect indicate that lower loading is associated with worse future patient-reported outcomes. *1* first half of stance phase, *2* second half of stance phase, *ADL* activities of daily living, *CI* confidence interval, *IR* internal rotation, *KAM* knee adduction moment, *KFM* knee flexion moment, *KOOS* Knee injury and Osteoarthritis Outcomes Score, *mGRF* medial ground reaction force, *QoL* quality of life, *Rec* recreation, *vGRF* vertical ground reaction force, ^1^limb symmetry index; ^2^side to side differences

Due to heterogeneity precluding meta-analysis, the evidence for the association between loading measures and future symptoms has been summarised using a narrative synthesis approach. Overall, there was inconsistent evidence for the longitudinal association between knee joint loading variables (as measured through vGRF, KFM, medial GRF, KAM) at 6 months to 2 years and future patient-reported outcomes at 6 months to 8 years (Fig. 7). However, when considering more specifically each biomechanical variable, lower vGRF at 6 months following ACLR was associated with worse patient-reported outcomes at 1 year [41, 45], but lower vGRF at 2 years post-ACLR was associated with better patient-reported outcomes at 10 years [43], suggesting time post-surgery may influence the association between mechanical loading and self-reported symptoms. Lower measures of frontal plane kinetics (KAM, medial GRF) at 6 months and 2 years post-ACLR were associated with better patient-reported outcomes over time [41, 42].

### Longitudinal Association Between Kinematic Measures and Future Structural and Symptom Outcomes

The longitudinal association between knee joint kinematics and future joint structure and symptoms was also inconsistent when synthesised narratively. One study found that a greater peak KFA during walking at 3 months post-ACLR was associated with better structural outcomes in the patellofemoral joint at 2 years post-ACLR [40]. A greater KFA during landing at 6 months was associated with worsening T1 rho relaxation times between 6 months and 3 years post-ACLR in the tibiofemoral joint [36], but a higher KFA during the loading response of gait at 1-year post-ACLR was related to worsening T1 rho and T2 relaxation times in the tibiofemoral joint at 1 and 2 years post-ACLR [38]. With regard to symptoms, one study found that a greater KFA during landing at the time of return to sport post-ACLR was associated with better future KOOS scores 2 years after return to sport [44], with another finding no relationship between KFA during walking at 2 years post-ACLR and a change in KOOS scores between 2 and 10 years [42]. Titchenal et al. [46] found that increasing anterior or lateral positions of the knee centre of rotation in the ACLR knee between 2 and 4 years were associated with worsening KOOS scores between 2–4 and 2–8 years.

## Discussion

This systematic review of 18 longitudinal studies demonstrated that altered knee joint biomechanics following surgery are not consistently associated with the onset and progression of structural and/or symptomatic outcomes relating to post-traumatic OA. One important finding from our meta-analysis indicates that underloading of the patellofemoral joint within the first year following ACLR is related to worsening future structural outcomes in trochlear cartilage.

### Underloading of the Patellofemoral Joint: A Potential Risk Factor for Future Post-Traumatic OA

Lower patellofemoral contact force/pressure between 3 months and 1 year may be associated with worsening future trochlear cartilage status at 1–5 years, with a medium effect size. This contrasts with what is typically observed with insidious-onset OA, where overloading is more closely linked to OA outcomes [12–14, 47–49]. This result was obtained from our meta-analysis (Fig. 2a) where different structural outcomes (i.e. cartilage quality from T2 mapping relaxation time, cartilage lesions from a semi-quantitative assessment) were pooled, and was reinforced by the findings of our narrative synthesis (Fig. 3).

Our findings in the patellofemoral joint are perhaps unsurprising when considering the properties of articular cartilage and its response post-injury. For example, articular cartilage is mechanosensitive and relies on appropriate stimuli and optimal joint loading for health [29, 50, 51]. This is an important consideration particularly following traumatic injury, when concomitant damage to knee cartilage is commonplace, with one study reporting 100% of patients sustained an MRI-detectable cartilage injury at the time of initial ACL injury [52]. Surgical interventions alone (e.g. ACLR, meniscal repair) do not fully restore pre-injury biomechanics [7, 8], and the early post-operative period likely contributes to a period of rest and subsequent underloading. This suboptimal loading on already compromised cartilage may exacerbate the impact of loading on structural outcomes and cartilage health. Furthermore, following traumatic injury, underloading may occur because of lower knee flexion angles, knee extensor moments, quadriceps forces and/or “quadriceps avoidance” strategies [53–55]. Lower knee flexion angles post-ACLR may reduce the contact area between the patella and the trochlear groove, potentially increasing patellofemoral joint (PFJ) stress by concentrating loads on smaller cartilage regions. While most studies use generalised contact area estimates to quantify PFJ stress [34, 54, 55], patient-specific measures derived from individual imaging could enhance accuracy by accounting for individual variations in joint geometry and kinematics. The combination of prolonged underloading and cartilage damage is associated with decreased chondrocyte activity [56], lower proteoglycan density [17] and increased catabolic activity in the cartilage [57], leading to thinner and softer cartilage more susceptible to the degenerative process [50]. Consequently, as the ability of the cartilage to withstand mechanical load decreases over time, even smaller external loads may exceed this threshold, effectively creating an overload scenario that accelerates degenerative processes [58]. Additionally, alterations in the knee extensor mechanism may disrupt patellar tracking, leading to a lateral displacement or tilt, which can redistribute loads to more vulnerable PFJ cartilage regions, creating focal regions of overloading. Although patellar maltracking has been shown to increase patellofemoral OA risk post-ACLR [59], no studies evaluating patellar tracking during dynamic tasks met the eligibility criteria for this review. This dynamic interplay between underloading, altered patellar tracking and reduced cartilage capacity highlights the importance of optimising loading post-injury to mitigate OA risk.

Our finding of an association between underloading and trochlear, but not patellar, cartilage health is consistent with other studies that demonstrate more pronounced early OA changes in trochlear cartilage following ACLR [60–64]. This may reflect the distinct properties of trochlear cartilage, as patellar cartilage is thicker [65, 66] and less affected by age-related thickness reduction [66]. Monitoring of trochlear cartilage composition early after surgery may therefore be the most sensitive approach for detecting early changes that contribute to the future development of post-traumatic OA.

### Longitudinal Relationship Between Mechanical Loading and Tibiofemoral OA is More Inconsistent

We found inconsistent evidence regarding the longitudinal relationship between biomechanics and future tibiofemoral structure over time. Variation in study populations (two studies assessed participants post-ACLR [31, 39] and one assessed following arthroscopic partial meniscectomy [32]), sample sizes (range 14–70), imaging modalities (e.g. MRI, X-ray), MRI sequences, structural outcomes and structural regions investigated across studies in our meta-analyses (Figs. 4a–b) may have influenced our results. For example, MRI outcomes, particularly T1 rho and T2 relaxation times, are more sensitive than radiographs for identification of early-stage OA [67]. Furthermore, one study stratified their participants into T1 rho high and T1 rho low groups (higher T1 rho values being indicative of worse cartilage health) and found that, although those with worse cartilage health had higher KFM at 6 months, both ACLR groups underloaded in comparison to uninjured controls [31], suggesting that joint underloading is present and thus may still have relevance to the onset of post-traumatic tibiofemoral OA. Our results from the included longitudinal studies appear to reflect findings from similar cross-sectional studies, where lower loading variables during walking were associated with both adverse [16–18, 56, 68] and beneficial [69] structural outcomes. Interestingly, in cohorts of insidious-onset knee OA, higher loading variables (e.g. KAM, KFM, varus thrust) are more consistently associated with OA onset and/or progression [12, 47, 48, 70]. Our results therefore appear to reinforce previous findings that gait-related risk factors for OA differ between traumatic and non-traumatic populations [16].

### Longitudinal Relationship Between Mechanical Loading and Future Symptoms

From our narrative synthesis of longitudinal studies (Fig. 7), lower measures of frontal plane kinetics (KAM, medial GRF) were associated with better future symptoms, regardless of their assessment timepoint post-surgery [41, 42]. Higher KAM and medial ground reaction force have been shown to be correlated with medial contact force [71, 72], which is, in turn, associated with worse symptoms in those with insidious-onset knee OA [13, 49, 73]. In contrast, when examining vGRF alone, a measure that can be easily and reliably replicated in clinical settings [74], our findings suggest that time post-surgery may influence the association between mechanical loading and future self-reported symptoms. For example, we found that underloading at 6 months post-ACLR may be a risk factor for worsening symptoms at 1 year [41, 45], but lower loading when assessed at 2 years post-surgery may be linked to better symptoms at 8–10 years [42, 43]. These findings need to be interpreted with caution given the small number of studies contributing data (*n* = 2 at 6 months and *n* = 2 at 2 years). Nevertheless, this is concurrent with previous research that has shown that symptomatic individuals less than 12 months post-ACLR demonstrate less vGRF at weight acceptance and propulsion phases of walking compared with asymptomatic individuals post-ACLR, but greater vGRF when assessed over 24 months post-surgery [75].

### Clinical Implications

Considering that the patellofemoral joint is most commonly affected by OA post-ACLR [61] and is associated with considerable patient burden [76], optimising patellofemoral loads post-operatively and addressing modifiable biomechanical alterations, such as increasing patellofemoral contact force and reducing medial force, may help to slow the onset and progression of post-traumatic patellofemoral OA. Force plates are readily accessible, easy to use, and have demonstrated validity and reliability for the assessment of vGRF during commonly used movement, strength and balance tests [74]. Although they do not directly measure patellofemoral contact force, force plates provide a practical measure of lower extremity mechanical loading post-surgery, enabling clinicians to monitor loading patterns at different timepoints. This information can be used to facilitate discussions concerning prognosis related to post-traumatic OA and individualise treatment to address specific identified deficits. Movement retraining and biofeedback can also be used to manipulate vGRF, restore optimal gait patterns and improve cartilage health [77]. In addition, commonly prescribed exercises can be altered to modify patellofemoral joint load [78], and treatment adjuncts, such as knee braces [79–85], footwear or insoles [86, 87], may also help to manipulate biomechanical factors and symptoms. Clinical trials are required to confirm the effectiveness of these approaches in preventing or slowing post-traumatic OA. [76, 88]

### Limitations

There are a number of limitations to consider when interpreting the results of this review. First, our search was restricted to articles published in English. This was because of the lack of resources available to translate. Second, included studies had small sample sizes (*n* = 14–70) with a varied risk of bias. No studies achieved a low risk of bias across all domains. Third, because only 3 of 12 studies investigating structural outcomes were included in each of the four meta-analyses, we were unable to quantitatively explore explanations for the large degree of heterogeneity in the included studies. Heterogeneity in biomechanical tasks, assessment timepoints and structural outcome measures further complicates interpretation of these pooled results, which should be interpreted with caution. These studies are also integrated into the narrative synthesis for a more comprehensive overview. Fourth, most of the included studies assessed walking biomechanics, and as such may not be generalisable to higher demand activities (e.g. drop jumps), where decreased knee flexion angles may pose greater risks. Fifth, the included studies used both conventional biomechanical measures (e.g. joint moments) and newer measures (e.g. contact force/stress), but advanced modelling techniques, such as finite element analysis, could offer deeper insights into joint loads and longitudinal cartilage changes. Sixth, in our narrative synthesis, while multiple reports from research groups provided valuable insights into biomechanical factors and future post-traumatic OA, some involved overlapping cohorts, as noted in Table 1. Readers should interpret these findings cautiously, as they may overemphasise specific populations. Seventh, 6 of the 18 studies across our review measured limb symmetry, which does not account for the fact that individuals post-ACLR also underload their contralateral uninvolved limb compared to uninjured controls [34]. Assessing limb symmetry alone may therefore give a misleading impression of recovery and return to full function of the injured limb, when in fact it does not reflect the overall impairment in both limbs when compared to uninjured controls [89]. Eighth, four studies reported change in biomechanical variables, which has the potential to be incorrectly interpreted as excessive magnitudes, when in reality the large change in value may be normalisation from a baseline measure of underloading to values similar to uninjured controls [31]. Ninth, while we investigated the isolated relationship between biomechanics and structure and symptoms, the factors influencing the development and progression of structural and symptomatic post-traumatic OA are complex and unlikely to be linked to any single factor (e.g. biomechanics) alone. Finally, although we intended to explore sex/gender differences in the associations between biomechanics and future structural and symptomatic outcomes, this analysis was not possible owing to a lack of sex/gender separated data in the included studies. For future research, we emphasise the importance of standardisation and consistency across methodologies and common language variables to improve comparability across future studies.

## Conclusions

In this systematic review, we found that underloading the patellofemoral joint up to 1-year post-surgery is linked with worse future structural outcomes in the patellofemoral joint. In general, we found inconsistent evidence regarding the longitudinal relationship between other biomechanical features and future tibiofemoral joint structure and patient-reported outcomes. Our findings suggest that the timing of assessment post-surgery may influence the relationship between mechanical loading and future symptoms, with lower vGRF at 6 months associated with better future symptoms. However, lower measures of frontal plane kinetics (KAM, medial ground reaction force) may lead to better future symptoms, regardless of the timepoint at which they were assessed. Our research highlights the complex interplay between knee joint loading, biomechanics, and the onset and progression of post-traumatic OA that should be considered in efforts to prevent and manage post-traumatic OA.

## Supplementary Information

Below is the link to the electronic supplementary material.

Supplementary file1 (DOCX 61 KB)

## Declarations

### Availability of Data and Material

Data are included in Table 1 and the supplementary tables. Further data are available from the corresponding author on reasonable request.
