# Supplementary material for: Are Altered Knee Joint Biomechanics Associated with Future Post-Traumatic Osteoarthritis Outcomes? A Systematic Review and Meta-Analysis of Longitudinal Studies
Source: Sports Med. 2025 Aug 5;55(10):2595–612. doi: 10.1007/s40279-025-02288-1 (PMC12513957; doi:10.1007/s40279-025-02288-1)
Supplement: Supplementary file 1 — Supplementary file1 (DOCX 61 KB) [file 40279_2025_2288_MOESM1_ESM.docx]

**Are Altered Knee Joint Biomechanics Associated with Future Post-Traumatic Osteoarthritis Outcomes? A Systematic Review and Meta-Analysis of Longitudinal Studies.**

Matthew Savage^1, 2^; Adam G Culvenor^1, 2^; Michael Hedger^1, 2^; April-Rose Matt^1, 2^; Michael JM O’Brien^1, 2^; Rachael M McMillan^3^; Alysha De Livera^1, 4^; Benjamin F Mentiplay^1, 2, 5^

^1^ La Trobe Sport and Exercise Medicine Research Centre, School of Allied Health, Human Services and Sport, La Trobe University, Melbourne, Victoria, Australia

^2^ Australian IOC Research Centre, La Trobe University, Melbourne, Victoria, Australia

^3^ School of Medicine, Deakin University, Waurn Ponds, Victoria, Australia

^4^ Mathematics and Statistics, School of Computing, Engineering and Mathematical Sciences, La Trobe University, Melbourne, Victoria, Australia

^5^ Sport and Exercise Science, School of Allied Health, Human Services and Sport, La Trobe University, Melbourne, Victoria, Australia

**Corresponding Author:**

**Dr Benjamin Mentiplay**

Senior Lecturer in Sport and Exercise Science

La Trobe Sport and Exercise Medicine Research Centre

La Trobe University

Email: [b.mentiplay@latrobe.edu.au](mailto:b.mentiplay@latrobe.edu.au); Phone: +61 3 9479 6417

Contents

[**Appendix A.** Search Strategy 2](#_Toc199358484)

[**Appendix B.** QUIPS tool 5](#_Toc199358485)

[**Appendix C.** Data used for meta-analysis 8](#_Toc199358486)

[**Appendix D.** Details of study exposure and outcome measures 10](#_Toc199358487)

[**Appendix E.** Risk of bias assessment of included studies. 13](#_Toc199358488)

# **Appendix A.** Search Strategy

**MEDLINE**

1. Knee/ or Knee Joint/ or Patellofemoral joint/ or Menisci, tibial/ or Patellofemoral Joint/ or Anterior Cruciate Ligament/ or Posterior Cruciate Ligament/ or Patella/
2. (knee or tibiofemoral or patellofemoral or "cruciate ligament" or ACL or PCL or "Collateral ligament" or MCL or LCL or Menisc* or Patella or Trochlea).tw,kf.
3. 1 or 2
4. Arthroscopy/ or Reconstructive Surgical Procedures/ or Anterior Cruciate Ligament Reconstruction/ or Posterior Cruciate Ligament Reconstruction/ or Meniscectomy/ or Osteotomy/)
5. (surg* or operati* or reconstruct* or resect* or repair or post operati* or post-operati* or postoperati* or Arthroscop* or Shav*).tw,kf
6. 4 or 5
7. Biomechanical Phenomena/ or Movement/ or Kinetics/
8. ("joint load" or kinetic* or kinematic* or "ground-reaction force" or "ground reaction force" or GRF or "knee adduction moment" or KAM or "knee flexion moment" or "transverse plane moment" or "external rotation" or valgus or varus or "knee moment" or biomechanic* or "contact force" or spatiotemporal or temporospatial).tw,kf.
9. 7 or 8
10. Osteoarthritis, Knee/ or Osteoarthritis / or Cartilage/ or Articular cartilage/ or Hyaline cartilage/
11. (osteoarthr* or OA or arthrosis* or gonarth* or "degenerative arthritis" or "joint space narrowing" or osteophyt* or cartilage or chondral or subchondral or osteochondral or "marrow oedema" or "marrow edema" or "marrow lesion" or BMO or BML).tw,kf
12. 10 or 11
13. Pain/ or "Quality of life"/ or Patient reported outcome measures/
14. (Pain or "visual analogue" or VAS or "numerical rating" or NRS or "western ontario and mcmaster" or WOMAC or "knee injury and osteoarthritis outcome score" or Koos or SF36 or sf-36 or "short form survey" or eq5d or eq-5d or EuroQoL or "euro qol" or "Cincinnati knee rating system" or "Lysholm Knee Score" or IKDC or "international knee documentation committee" or ACL-QOL or "anterior cruciate ligament quality of life questionnaire" or kqol-26 or "kqol 26" or "knee quality of life" or LEFS or "Lower extremity functional scale" or KOS or "knee outcome survey" or TSK or "tampa scale of kinesiophobia").tw,kf.
15. 13 or 14
16. 12 or 15
17. 3 and 6 and 9 and 16

**EMBASE**

1. Knee/ or Knee Joint/ or Patellofemoral joint/ or Menisci, tibial/ or Patellofemoral Joint/ or Anterior Cruciate Ligament/ or Posterior Cruciate Ligament/ or Patella/ or Femur/ or Tibia/
2. (knee or tibiofemoral or patellofemoral or "cruciate ligament" or ACL or PCL or "Collateral ligament" or MCL or LCL or Menisc* or Patella or Trochlea).tw
3. 1 or 2
4. Arthroscopy/ or Reconstructive Surgical Procedures/ or Anterior Cruciate Ligament Reconstruction/ or Posterior Cruciate Ligament Reconstruction/ or Meniscectomy/ or Osteotomy/
5. (surg* or operati* or reconstruct* or resect* or repair or post operati* or post-operati* or postoperati* or Arthroscop* or Shav*).tw
6. 4 or 5
7. Biomechanical Phenomena/ or Movement/ or Kinetics/
8. ("joint load" or kinetic* or kinematic* or "ground-reaction force" or "ground reaction force" or GRF or "knee adduction moment" or KAM or "knee flexion moment" or "transverse plane moment" or "external rotation" or valgus or varus or "knee moment" or biomechanic* or "contact force" or spatiotemporal or temporospatial).tw
9. 7 or 8
10. Osteoarthritis, Knee/ or Osteoarthritis / or Cartilage/ or Articular cartilage/ or Hyaline cartilage/
11. (osteoarthr* or OA or arthrosis* or gonarth* or "degenerative arthritis" or "joint space narrowing" or osteophyt* or cartilage or chondral or subchondral or osteochondral or "marrow oedema" or "marrow edema" or "marrow lesion" or BMO or BML).tw,kf
12. 10 or 11
13. Pain/ or "Quality of life"/ or Patient reported outcome measures/
14. (Pain or "visual analogue" or VAS or "numerical rating" or NRS or "western ontario and mcmaster" or WOMAC or "knee injury and osteoarthritis outcome score" or Koos or SF36 or sf-36 or "short form survey" or eq5d or eq-5d or EuroQoL or "euro qol" or "Cincinnati knee rating system" or "Lysholm Knee Score" or IKDC or "international knee documentation committee" or ACL-QOL or "anterior cruciate ligament quality of life questionnaire" or kqol-26 or "kqol 26" or "knee quality of life" or LEFS or "Lower extremity functional scale" or KOS or "knee outcome survey" or TSK or "tampa scale of kinesiophobia").tw
15. 13 or 14
16. 12 or 15
17. 3 and 6 and 9 and 16

**CINAHL + SPORTDISCUS**

1. TI ( knee or tibiofemoral or patellofemoral or "cruciate ligament" or ACL or PCL or "Collateral ligament" or MCL or LCL or Menisc* or Patella or Trochlea ) OR AB ( knee or tibiofemoral or patellofemoral or "cruciate ligament" or ACL or PCL or "Collateral ligament" or MCL or LCL or Menisc* or Patella or Trochlea )
2. TI ( surg* or operati* or reconstruct* or resect* or repair or post operati* or post-operati* or postoperati* or Arthroscop* or Shav ) OR AB ( surg* or operati* or reconstruct* or resect* or repair or post operati* or post-operati* or postoperati* or Arthroscop* or Shav )
3. S1 and S2
4. TI ( "joint load" or kinetic* or kinematic* or "ground-reaction force" or "ground reaction force" or GRF or "knee adduction moment" or KAM or "knee flexion moment" or "transverse plane moment" or "external rotation" or valgus or varus or "knee moment" or biomechanic* or "contact force" or spatiotemporal or temporospatial ) OR AB ( "joint load" or kinetic* or kinematic* or "ground-reaction force" or "ground reaction force" or GRF or "knee adduction moment" or KAM or "knee flexion moment" or "transverse plane moment" or "external rotation" or valgus or varus or "knee moment" or biomechanic* or "contact force" or spatiotemporal or temporospatial )
5. S3 AND S4
6. TI ( osteoarthr* or OA or arthrosis* or gonarth* or "degenerative arthritis" or "joint space narrowing" or osteophyt* or cartilage or chondral or subchondral or osteochondral or "marrow oedema" or "marrow edema" or "marrow lesion" or BMO or BML ) OR AB ( osteoarthr* or OA or arthrosis* or gonarth* or "degenerative arthritis" or "joint space narrowing" or osteophyt* or cartilage or chondral or subchondral or osteochondral or "marrow oedema" or "marrow edema" or "marrow lesion" or BMO or BML )
7. TI ( Pain or "visual analogue" or VAS or "numerical rating" or NRS or "western ontario and mcmaster" or WOMAC or "knee injury and osteoarthritis outcome score" or Koos or SF36 or sf-36 or "short form survey" or eq5d or ed-5d or EuroQoL or "euro qol" or "Cincinnati knee rating system" or "Lysholm Knee Score" or IKDC or "international knee documentation committee" or ACL-QOL or "anterior cruciate ligament quality of life questionnaire" or kqol-26 or "kqol 26" or "knee quality of life" or LEFS or "Lower extremity functional scale" or KOS or "knee outcome survey" or TSK or "tampa scale of kinesiophobia" ) OR AB ( Pain or "visual analogue" or VAS or "numerical rating" or NRS or "western ontario and mcmaster" or WOMAC or "knee injury and osteoarthritis outcome score" or Koos or SF36 or sf-36 or "short form survey" or eq5d or ed-5d or EuroQoL or "euro qol" or "Cincinnati knee rating system" or "Lysholm Knee Score" or IKDC or "international knee documentation committee" or ACL-QOL or "anterior cruciate ligament quality of life questionnaire" or kqol-26 or "kqol 26" or "knee quality of life" or LEFS or "Lower extremity functional scale" or KOS or "knee outcome survey" or TSK or "tampa scale of kinesiophobia" )
8. S6 OR S7
9. S5 AND S8

**SCOPUS**

1. (knee or tibiofemoral or patellofemoral or "cruciate ligament" or ACL or PCL or "Collateral ligament" or MCL or LCL or Menisc* or Patella or Trochlea)
2. (surg* or operati* or reconstruct* or resect* or repair or post operati* or post-operati* or postoperati* or Arthroscop* or Shav*)
3. 1 and 2
4. ("joint load" or kinetic* or kinematic* or "ground-reaction force" or "ground reaction force" or GRF or "knee adduction moment" or KAM or "knee flexion moment" or "transverse plane moment" or "external rotation" or valgus or varus or "knee moment" or biomechanic* or "contact force" or spatiotemporal or temporospatial)
5. 3 and 4
6. (osteoarthr* or OA or arthrosis* or gonarth* or "degenerative arthritis" or "joint space narrowing" or osteophyt* or cartilage or chondral or subchondral or osteochondral or "marrow oedema" or "marrow edema" or "marrow lesion" or BMO or BML)
7. (Pain or "visual analogue" or VAS or "numerical rating" or NRS or "western ontario and mcmaster" or WOMAC or "knee injury and osteoarthritis outcome score" or Koos or SF36 or sf-36 or "short form survey" or eq5d or ed-5d or EuroQoL or "euro qol" or "Cincinnati knee rating system" or "Lysholm Knee Score" or IKDC or "international knee documentation committee" or ACL-QOL or "anterior cruciate ligament quality of life questionnaire" or kqol-26 or "kqol 26" or "knee quality of life" or LEFS or "Lower extremity functional scale" or KOS or "knee outcome survey" or TSK or "tampa scale of kinesiophobia"))
8. 6 or 7
9. 5 and 8
10. AND (LIMIT-TO ( EXACTKEYWORD,"Human" ) OR LIMIT-TO (EXACTKEYWORD,"Humans")) AND (LIMIT-TO ( LANGUAGE,"English" ))

# **Appendix B.** QUIPS tool

| **Author and year of publication:** |  | | |
| --- | --- | --- | --- |
| **Reviewer:** |  | | |
| **Biases** | **Study methods and comments** | **Rating of reporting (yes, partial, no, or unsure)** | **Rating of risk of bias (high, moderate, or low)** |
| 1. **Study Participation** | *To judge the risk of selection bias. Does the study sample represent the population of interest?* |  |  |
| Adequate participation in the study by eligible persons |  |  |  |
| Description of the source population or population of interest |  |  |  |
| Description of the baseline study sample |  |  |  |
| Adequate description of the sampling frame and recruitment |  |  |  |
| Adequate description of the period and place of recruitment |  |  |  |
| 1. **Study Attrition** | *To judge the risk of attrition bias. Does the data from participants not lost to follow up accurately represent the sample?* |  |  |
| Adequate response rate for study participants |  |  |  |
| Description of attempts to collect information on participants who dropped out |  |  |  |
| Reasons for loss to follow-up are provided |  |  |  |
| Adequate description of participants lost to follow-up |  |  |  |
| There are no important differences between participants who  completed the study and those who did not |  |  |  |
| 1. **Prognostic Factor Measurement** | *To judge the risk of measurement bias. Is the risk factor similarly measured for all participants?* |  |  |
| A clear definition or description of the risk factor is provided |  |  |  |
| Method of risk factor measurement is adequately valid and reliable |  |  |  |
| Continuous variables are reported or appropriate cut points are used |  |  |  |
| The method and setting of measurement of risk factor is the same for all study participants |  |  |  |
| Adequate proportion of the study sample has complete data for the risk factor |  |  |  |
| Appropriate methods of imputation are used for missing risk factor data |  |  |  |
| 1. **Outcome Measurement** | *To judge the risk of bias related to the measurement of outcome. Is the outcome similarly measured for all participants?* |  |  |
| A clear definition of the outcome is provided |  |  |  |
| Method of outcome measurement used is adequately valid and reliable |  |  |  |
| The method and setting of outcome measurement is the same for all study participants |  |  |  |
| 1. **Study Confounding** | *To judge the risk of bias due to confounding factors. Are important potential confounding factors accounted for?* |  |  |
| All important confounders are measured |  |  |  |
| Clear definitions of the important confounders measured are provided |  |  |  |
| Measurement of all important confounders is adequately valid and reliable |  |  |  |
| The method and setting of confounding measurement are the same for all study participants |  |  |  |
| Appropriate methods are used if imputation is used for missing confounder data |  |  |  |
| Important potential confounders are accounted for in the study design |  |  |  |
| Important potential confounders are accounted for in the analysis |  |  |  |
| 1. **Statistical Analysis and Reporting** | *To judge the risk of bias related to the statistical analysis and presentation of results. Is the analysis appropriate, and are all primary outcomes reported?* |  |  |
| Sufficient presentation of data to assess the adequacy of the analytic strategy |  |  |  |
| Strategy for model building is appropriate and is based on a conceptual framework or model |  |  |  |
| The selected statistical model is adequate for the design of the study |  |  |  |
| There is no selective reporting of results |  |  |  |

# **Appendix C.** Data used for meta-analysis

| **Study** | **Exposure** | **Effect Measure for meta-analysis** | **Reported Effect Measure** | **Sample Size** | **Available data** |
| --- | --- | --- | --- | --- | --- |
| **Patellofemoral Joint** | | | | | |
| Liao et al. [34] | Patellofemoral contact force | Pearson’s correlation coefficient | Pearson’s correlation coefficient | 32 | Trochlea: r =-0.47  Patella: r = -0.49 |
| Schache et al. [35] | Patellofemoral contact force | Pearson’s correlation coefficient | Risk ratio (95% CI) | 32 | Raw data used to calculate point biserial correlations |
| Williams et al. [40] | Patellofemoral contact force | Pearson’s correlation coefficient | Pearson’s correlation coefficient | 30 | Trochlea: r =-0.549  Patella: r =-0.089 |
| **Tibiofemoral Joint** | | | | | |
| Evans-Pickett et al. [31] | Peak knee flexion moment  Peak knee adduction moment | Standardised mean difference | Cohen's d | 26 | Peak KFM: Cohen’s d = 0.37  95% CI = 0.05 to 0.69  Peak KAM: Cohen’s d = -0.67  95% CI = -1.00 to -0.34 |
| Hall et al. [32] | Peak knee flexion moment  Peak knee adduction moment | Standardised mean difference | Odds ratio | 70 | Peak KFM:  OR = 1.07 95% CI = 0.67 to 1.72  Peak KAM: OR = 2.4  95% CI = 0.98 to 5.89  Odds ratio re-expressed as standardised mean difference following Cochrane Handbook guidelines (chapter 15) [26] |
| Wellsandt et al. [39] | Peak knee flexion moment  Peak knee adduction moment | Standardised mean difference | Mean (SD) in each group | 14 | Peak KFM: OA group mean = 0.35 OA group SD = 0.14  No OA group mean = 0.46 No OA group SD = 0.13  Peak KAM: OA group mean = 0.26 OA group SD = 0.08  No OA group mean = 0.29 No OA group SD = 0.08  Means (SD) in each group was converted to standardised mean difference using ‘metafor’ package for R |

MA = meta-analysis; KFM = knee flexion moment; KAM = knee adduction moment; r = Pearson’s correlation coefficient; RR = risk ratio; SMD = standardised mean difference; OR = odds ratio; SD = standard deviation; 95% CI = 95% confidence interval; OA = osteoarthritis.

# **Appendix D.** Details of study exposure and outcome measures

| Study | Task | Biomechanical metrics | Biomechanical definition | Baseline biomechanics timepoint | Outcomes | Follow-up outcome timepoint |
| --- | --- | --- | --- | --- | --- | --- |
| Structure | | | | | | |
| Capin et al. [29] | Walking preferred speed | Peak KFM^β^ | Peak KFM (0-100% stance) | 3.3 ± 0.6 months | **T2 relaxation time**  Trochlea^β^ | 6.3 ± 0.5 months |
|  |  | Peak quadriceps muscle forces^β^ | Peak quadriceps muscle forces (0-50% of stance) |  |  |  |
|  |  |  | |  |  |  |
|  |  | Peak KFA ^β^ | Peak KFA (~0-25% of stance) |  |  |  |
|  |  | Knee flexion excursion^β^ | Knee flexion excursion from initial contact to peak knee flexion (~0-25% of stance) |  |  |  |
|  |  | Knee extension excursion^β^ | Knee extension excursion from peak knee flexion to peak knee extension (midstance) |  |  |  |
| Erhart-Hledik et al. [30] | Walking preferred speed | Total joint moment 1 | Total joint moment 1 = $\surd$KFM^2^+KAM^2^+KRM^2^ (0-50% stance) | Δ 2.2 ± 0.3 to 7.7 ± 0.7 years | **Cartilage thickness ratio (medial:lateral)**  Femur | Δ 2.2 ± 0.3 to 7.7 ± 0.7 years |
|  |  | Percentage contribution of KFM to total joint moment 1 | KFM/KAM % contribution to TJM at TJM1 (0-50% stance) |  |  |  |
|  |  | Percentage contribution of KAM to total joint moment 1 |  |  |  |  |
| Evans-Pickett et al. [31] | Walking preferred speed | KFM | Performed functional waveform gait analysis for the entire stance phase to detect between-group differences (high vs low T1rho) for KFM and KAM, with significant differences at:  KFM (13-20% stance phase)  KAM (11-47% stance phase and 59-95% stance phase) | 6 months | **T1rho**  Femur  Tibia | 12 months |
|  |  | KAM |  |  |  |  |
| Hall et al.^ϕ^[32] | Walking preferred speed  Walking fast-pace | Peak KFM | Peak KFM (0-50% of stance) | 3 months | **Cartilage volume**  Medial tibia  Patella  **Cartilage defects**  Medial tibiofemoral  Patella | Δ 3 months –  2.06 ± 0.12 years |
|  |  | Peak KAM | Peak KAM (0-50% of stance) |  |  |  |
|  |  | KAM impulse | KAM impulse positive (0-100% of stance) |  |  |  |
| Kumar et al. [33] | Walking fixed speed 1.35m/s | Peak KAM | Peak KAM (0-50% of stance) | Δ pre-op –  6 months  Δ 6 months –  1 year | **T1 rho**  Medial femur  Medial tibia  **T2 relaxation time**  Medial femur  Medial tibia | Δ pre-op –  6 months  Δ 6 months – 1 year |
|  |  | KAM impulse | KAM impulse positive (0-100% of stance) |  |  |  |
| Liao et al. [34] | Walking fixed speed 1.3m/s | Peak PFJ contact pressure | Peak PFJ contact pressure (0-50% of stance) | 6 months | **T2 relaxation time**  Patella  Trochlea | 3 years |
| Schache et al. [35] | Forward hop | Peak PFJ contact force | Peak PFJ contact force (0-100% landing phase, defined as initial contact to peak knee flexion) | 14 ± 2 months | **Cartilage lesions**  Patella  Trochlea | Δ 14 ± 2 to 62 ± 2 months |
|  |  | Time to peak PFJ contact force | Time to peak PFJ contact (% of landing phase) |  |  |  |
|  |  | Impulse of PFJ contact force | Impulse of PFJ contact force (0-100% landing phase) |  |  |  |
| Shimizu et al. [36] | Drop jump | Peak KFM^α^ | Peak KFM (0-100% landing phase, defined as initial contact to toe-off) | 6 months  Δ 6 months –  3 years | **T1 rho**  Medial femur  Medial tibia  cMT | Δ 6 months – 3 years |
|  |  | KFM impulse^α^ | KFM impulse (0-100% landing phase) |  |  |  |
|  |  | Peak vGRF^α^ | Peak vGRF (0-50% landing phase) |  |  |  |
|  |  |  |  |  |  |  |
|  |  | Peak KFA^α^ | Peak KFA (0-100% landing phase) |  |  |  |
| Shimizu et al. [37] | Drop jump | Peak KFM | Peak KFM (0-100% landing phase, defined as initial contact to toe-off) | 6 months | **T1 rho**  PHMED meniscus  **T2 relaxation time**  PHMED meniscus | Δ 6 months – 3 years |
|  |  | Peak vGRF | Peak vGRF (0-50% landing phase) |  |  |  |
| Teng et al. [38] | Walking fixed speed 1.3m/s | Peak KFM | Peak KFM (loading response, defined as initial contact to first peak KFA) | 6 months  1 year | **T1 rho**  Medial femur  Medial tibia  **T2 relaxation time**  Medial femur  Medial tibia | Δ pre-op –  1 year  Δ pre-op –  2 years |
|  |  | Peak vGRF | Peak vGRF (loading response) |  |  |  |
|  |  |  | |  |  |  |
|  |  | Peak KFA | Peak KFA (loading response) |  |  |  |
| Wellsandt et al. [39] | Walking preferred speed | Peak medial compartment contact force^β^ | Peak medial compartment force (0-50% of stance) | 6 months  1 year  2 years | **XR (KL)**  OA or no OA (medial compartment TFJ) | 5 years |
|  |  | Peak KFM^β^ | Peak KFM (0-100% stance) |  |  |  |
|  |  | Peak KAM^β^ | Peak KAM (0-100% stance) |  |  |  |
|  |  | KAM Impulse^β^ | KAM Impulse (0-100% stance) |  |  |  |
| Williams et al. [40] | Walking preferred speed | Peak PFJ contact force | NR | 3.2 ± 0.6 months | **T2 relaxation time**  Patella  Trochlea  (total, deep and superficial cartilage) | 24.6 ± 1.4 months |
|  |  | Peak KFM | NR |  |  |  |
|  |  |  | |  |  |  |
|  |  | Peak KFA | NR |  |  |  |
| Symptoms | | | | | |  |
| Azus et al. [41] | Walking fixed speed 1.33m/s | Peak KFM 2 | Peak KFM (50-100% stance) | 6 months | KOOS pain  KOOS symptoms | 1 year  Δ 6 months - 1 year |
|  |  | KFM impulse 2 | KFM impulse (50-100% stance) |  |  |  |
|  |  | Peak vGRF 2 | Peak vGRF (50-100% stance) |  |  |  |
|  |  | Medial peak GRF 2 | Medial peak GRF (50-100% stance) |  |  |  |
|  |  | Medial GRF impulse 1 | Medial GRF impulse (0-50% stance) |  |  |  |
|  |  | Medial GRF impulse 2 | Medial GRF impulse (50-100% stance) |  |  |  |
| Erhart-Hledik et al. [42] | Walking preferred speed | Peak KFM^α^ | Peak KFM (0-50% stance) | 2.2 ± 0.3 years | KOOS pain  KOOS QoL | Δ 2.2 ± 0.3 to 7.7 ± 0.7 years |
|  |  | Peak KAM^α^ | Peak KAM (0-50% stance) |  |  |  |
|  |  | Peak knee internal rotation moment^α^ | Peak knee internal rotation moment (50-100% stance) |  |  |  |
|  |  |  | |  |  |  |
|  |  | Average KFA^α^ | Average KFA (0-100% stance) |  |  |  |
|  |  | Average knee varus-valgus angle^α^ | Average knee varus-valgus angle (0-100% stance) |  |  |  |
|  |  | Average knee internal-external rotation angle^α^ | Average knee internal-external rotation angle (0-100% stance) |  |  |  |
|  |  | Average anterior femoral displacement relative to the tibia^α^ | Average anterior femoral displacement relative to the tibia (0-100% stance) |  |  |  |
| Erhart-Hledik et al. [43] | Walking preferred speed | Peak vGRF^α^ | Peak vGRF (0-50% stance) | 2.2 ± 0.3 | KOOS pain  KOOS symptoms  KOOS ADL  KOOS sport/rec  KOOS QoL | Δ 2.2 ± 0.3 to 10.5 ± 0.9 years |
| Ithurburn et al. [44] | Single leg drop landing | Knee flexion excursion ^α^ | Knee flexion maximum minus minimum (0-100% landing phase, defined as initial contact to lowest point of body CoM) | At time of RTS | KOOS pain  KOOS ADL  KOOS QoL | 2 years after RTS |
|  |  | Peak trunk flexion ^α^ | Peak trunk flexion (0-100% landing phase) |  |  |  |
| Pietrosimone et al. [45] | Walking preferred speed | Peak vGRF ^α^ | Peak vGRF (0-50% stance) | 6 months | KOOS pain  KOOS symptoms  KOOS ADL  KOOS sport/rec  KOOS QoL | 1 year |
|  |  | Instantaneous vGRF loading rate ^α^ | Peak of the first derivative of force-time curve (0-50% stance) |  |  |  |
| Titchenal et al. [46] | Walking preferred speed | Knee centre of rotation coordinates (AP) | First projecting lines coincident with the transepicondylar  axis of the femur onto the transverse plane of the tibia  using the coordinate transformation matrix, relating the  femoral and tibial anatomic coordinate systems for every  frame of motion capture during the stance phase. The AP and ML coordinates were calculated by solving the least squares intersection of the projected lines. | Δ 2-4 years | KOOS pain  KOOS symptoms  KOOS QoL | Δ 2.19 ± 0.31 to 4.34 ± 0.29 years  Δ 2.19 ± 0.31 to 7.92 ± 0.57 years |
|  |  | Knee centre of rotation coordinates (ML) |  |  |  |  |

vGRF = vertical ground reaction force; Medial GRF = medial ground reaction force; KFM = knee flexion moment; 1 = first half of stance phase; 2 = second half of stance phase; Δ = change in; XR = X-Ray; KL = Kellgren-Lawrence; KOOS = Knee Injury and Osteoarthritis Outcome Score, KFA = knee flexion angle; KAM = knee adduction moment; QoL = quality of life; RTS = return to sport; PFJ = patellofemoral joint; OA = osteoarthritis; TFJ = tibiofemoral joint; ~ = approximately; KRM = knee rotation moment; cMT = central medial tibia; CoM = centre of mass; AP = anterior-posterior; ML = medial-lateral; NR = not reported.

^α^ = Side to Side Difference/Limb Symmetry Index; ^β^ = limb value and Side to Side Difference; **^ϕ^** = arthroscopic partial meniscectomy (the other included studies are all from participants post-anterior cruciate ligament reconstruction).

# **Appendix E.** Risk of bias assessment of included studies.

|  | **Study Participation** | **Study Attrition** | **Prognostic Factor** | **Outcome Measurement** | **Confounding** | **Analysis** |
| --- | --- | --- | --- | --- | --- | --- |
| **Structure** | | | | | | |
| Capin et al. [29] | Medium | High | Low | Low | Medium | Low |
| Erhart-Hledik et al. [30] | Medium | Medium | Low | Low | Medium | Medium |
| Evans-Pickett et al. [31] | Medium | High | Low | Low | Medium | Low |
| Hall et al. [32] | Medium | Medium | Low | Low | Low | Low |
| Kumar et al. [33] | Low | High | Low | Low | Medium | Low |
| Liao et al. [34] | Low | Medium | Low | Low | Medium | Medium |
| Schache et al. [35] | High | Medium | Low | Low | Low | Low |
| Shimizu et al. [36] | Medium | Medium | Low | Low | Low | Medium |
| Shimizu et al. [37] | Medium | Medium | Low | Low | Low | Medium |
| Teng et al. [38] | Low | High | Low | Low | Low | Low |
| Wellsandt et al. [39] | Medium | Medium | Low | Low | Medium | Low |
| Williams et al. [40] | High | Medium | Low | Low | Medium | Low |
| **Symptoms** | | | | | | |
| Azus et al. [41] | Medium | Medium | Low | Low | Low | Low |
| Erhart-Hledik et al. [42] | Medium | High | Low | Low | High | Medium |
| Erhart-Hledik et al. [43] | Medium | High | Low | Low | Low | Low |
| Ithurburn et al. [44] | Low | Medium | Low | Low | Low | Low |
| Pietrosimone et al. [45] | Low | Medium | Low | Low | Medium | Low |
| Titchenal et al. [46] | Medium | High | Low | Medium | High | Medium |
